# Is There a Role for Probiotics in the Prevention of Preterm Birth?

**DOI:** 10.3389/fimmu.2015.00062

**Published:** 2015-02-17

**Authors:** Siwen Yang, Gregor Reid, John R. G. Challis, Sung O. Kim, Gregory B. Gloor, Alan D. Bocking

**Affiliations:** ^1^Department of Physiology, Obstetrics and Gynecology, University of Toronto, Toronto, ON, Canada; ^2^Lunenfeld Tanenbaum Research Institute, Mount Sinai Hospital, Toronto, ON, Canada; ^3^Department of Microbiology and Immunology, Western University, London, ON, Canada; ^4^Lawson Health Research Institute, London, ON, Canada; ^5^Department of Obstetrics and Gynecology, The University of Western Australia, Perth, WA, Australia; ^6^Department of Biochemistry, Western University, London, ON, Canada

**Keywords:** probiotics, preterm birth, infection and inflammation, cytokines, vaginal microbiome, bacterial vaginosis

## Abstract

Preterm birth (PTB) continues to be a global health challenge. An over-production of inflammatory cytokines and chemokines, as well as an altered maternal vaginal microbiome has been implicated in the pathogenesis of inflammation/infection-associated PTB. *Lactobacillus* represents the dominant species in the vagina of most healthy pregnant women. The depletion of *Lactobacillus* in women with bacterial vaginosis (BV) has been associated with an increased risk of PTB. It remains unknown at what point an aberrant vaginal microbiome composition specifically induces the cascade leading to PTB. The ability of oral or vaginal lactobacilli probiotics to reduce BV occurrence and/or dampen inflammation is being considered as a means to prevent PTB. Certain anti-inflammatory properties of lactobacilli suggest potential mechanisms. To date, clinical studies have not been powered with sufficiently high rates of PTB, but overall, there is merit in examining this promising area of clinical science.

## Introduction

The etiology of preterm birth (PTB) is multifactorial: 50% of the cases are idiopathic while 20–40% are disease specific or medically indicated deliveries such as pre-eclampsia or fetal growth restriction (FGR), which require delivery (1, 2). The remaining 25–30% of PTB can be attributed to intrauterine infection and/or inflammation (1, 2). Microorganisms can invade the uterus through the fallopian tube in a retrograde fashion from the abdominal cavity, hematogenously via the placenta or ascending through the cervix and vagina (3).

## Mechanism of Inflammation and Infection-Associated Preterm Labor

Microorganisms can reach the maternal intrauterine tissues through any mucosal surface and secrete phospholipase A2 to act on membrane phospholipids and form unesterified arachidonic acid (AA). The AA is converted into endoperoxide products and subsequently into primary prostaglandins (PGs; PGE2, PGF2a) by PGH synthase-2 and isomerases, respectively. Alternatively, some microbes secrete endotoxins, such as lipopolysaccharides (LPS), which specifically bind toll-like receptor 4 (TLR4) and activate the nuclear factor κ light-chain-enhancer of activated B cells (NFkB) pathway to induce pro-inflammatory cytokine and chemokine gene expression in the intrauterine tissues (amnion, chorion, and decidua), macrophages, and endothelial cells (4, 5). Pro-inflammatory cytokines interact with each other as well as with PGs in a feed-forward cascade, hence amplifying the inflammatory response (6, 7). Furthermore, pro-inflammatory cytokines enhance the expression of matrix metalloproteinase (MMPs), which are zinc-dependent enzymes that catalyze the degradation of collagen constituted-extracellular matrix of the cervix, fetal membrane, placenta, and uterus (8–11). Elevated levels of MMP-9 in the maternal plasma, and MMP-3 and MMP-8 in the amniotic fluid are associated with preterm labor (PTL) and/or microbial invasion of the amniotic cavity (11–13).

Bacteria and viruses can also cross an intact chorioamniotic membrane and induce intra-amniotic inflammation, a condition termed the fetal inflammatory response syndrome (FIRS). Elevated interleukin (IL)-6 and LPS-binding proteins are observed in the umbilical cord blood in FIRS-affected preterm neonates (14–17). Pathogenic microorganisms such as *Ureaplasma urealyticum* and *Mycoplasma hominis* have been isolated from the umbilical cord blood of very preterm newborns (18). Intrauterine infection can also lead to activation of the fetal hypothalamic–pituitary–adrenal (HPA) axis giving rise to increased cortisol biosynthesis and decreased metabolism of maternal cortisol to inactive cortisone by 11β-hydroxysteroid dehydrogenase-2 in the placenta (19). Sustained stimulation of placental corticotropin releasing hormone by fetal cortisol leads to an increase in PG production (20). PG in turn promotes a positive feed-forward loop that comprise an increase in the expression and production of gap junctions such as connexin 43 and pro-inflammatory cytokines including IL-6 and tumor necrosis factor alpha (TNFα) (20). Together, they promote synchronous and forceful myometrial contractions and PTL.

In short, microbes are well known for their involvement in PTL. In order to understand the origins of these organisms, studies have been undertaken on many sites in the reproductive tract, particularly the vagina.

## Altered Vaginal Microbiome and PTB

The vaginal microbiota composition is dynamic throughout a woman’s life. Before puberty, it is dominated by anaerobic bacteria (21). Rising estrogen levels at puberty lead to an increase in mucosal glycogen production whose metabolized substrates support vaginal colonization with lactobacilli (21, 22). This is one reason for the vagina to be highly colonized by lactobacilli during reproductive years and pregnancy (23). At menopause, lactobacilli abundance decreases coinciding with a reduction in circulating estrogen (24–26).

Gram-positive lactobacilli are facultative anaerobic bacteria, whose adherence to the vaginal mucosal epithelia appears to form an important line of defense against pathogens (27). There is no definitive “normal” vaginal microbiota, but in the vast majority of pregnant healthy women, lactobacilli dominate (23, 28, 29). Several important aspects of the vaginal microbiota have been uncovered recently, particularly by sequencing PCR-amplified universal 16S ribosomal DNA (rDNA): (1) the healthy vaginal microbiota is dominated by a few *Lactobacillus* species (30); (2) the detection of *Lactobacillus iners*, *Atopobium vaginae*, and bacterial vaginosis-associated bacteria 1, 2, and 3 (BVAB), is apparent in women with BV (30–32). Due to some variations within sequencing techniques, selection of suitable PCR primers, and sufficient depth, future studies may yet reveal more important profiles of healthy versus infected women (33, 34).

Although relatively few 16S DNA studies have been used with samples from pregnant women, indications are that the microbiota does fluctuate during this time. Some researchers have suggested that there are up to five different community state types (CSTs) of bacteria, clusters generated based on similarity in vaginal bacterial composition, in asymptomatic pregnant and non-pregnant women (23, 35). Three of the CSTs (I, II, III) are dominated by *Lactobacillus*, namely *L. iners, L. crispatus*, and *L. jensenii* and/or *L. gasseri*. Two others, CST IV-A and CST IV-B have low relative abundance of *Lactobacillus* spp. and are composed of *Peptoniphilus*, *Anaerococcus*, *Corynebacterium*, *Finegoldia*, and *Prevotella* (CST IV-A), and *Atopobium, Sneathia*, *Gardnerella*, *Ruminococcaceae*, *Parvimonas*, and *Mobiluncus* (CST IV-B) (23). Such studies have suggested that the vaginal microbiota composition of pregnant women has a higher abundance of *L. vaginalis, L. crispatus, L. gasseri*, and *L. jensenii*, but lower CST IV-B bacteria, and is more stable than non-pregnant women (23, 28), with *L. crispatus*, promoting stability (36). This remains to be verified, but it may be due to hormonal changes. With advancing gestational age, the relative abundance of *Lactobacillus* spp. increases while that of anaerobe or strict-anaerobe microbial species decreases (37).

Bacterial vaginosis is essentially a polymicrobial dysbiosis, characterized by an alteration in the endogenous vaginal microflora with an absent or decreased proportion of lactobacilli and dominance of *G. vaginalis, Prevotella bivia, Mobiluncus sp., Mycoplasma hominis*, and *A. vaginae* (23, 35, 38, 39). Aerobic vaginitis (AE) is an inflammatory condition in which organisms, such as *Escherichia coli* and *Staphylococcus aureus* dominate (40). In many clinical units, the diagnosis of BV involves using a Gram stain Nugent scoring system with or without the Amsel criteria (a vaginal pH >4.5, an amine fishy odor when vaginal fluid is mixed with potassium chloride, the presence of clue cells) (41, 42). A Nugent score of 7–10 seen microscopically as a near absence of rod shaped lactobacilli and high abundance of pathogenic morphotypes is considered BV (42). However, the reliability of the Nugent score has recently been questioned (29). Indeed, sequencing of the vaginal microbiota of women with BV reveals a diverse array of bacteria, including the presence of *L. iners* (32, 43, 44). Improvement in diagnostic accuracy for BV can be accomplished by using a DNA level of ≥10^9^ copies/mL for *G. vaginalis* and ≥10^8^ copies/mL for *A. vaginae* (45).

The prevalence of BV can vary between populations, but it remains common during pregnancy, where it is associated with a 40% increase in the risk of PTB (46). Women with an abnormal vaginal flora in their first trimester of pregnancy have a higher risk of delivering preterm (39). Although an earlier Cochrane Review (47) suggested that antibiotic treatment of abnormal vaginal flora (intermediate flora or BV) before 20 weeks of gestation may reduce the risk of PTB, a recent Cochrane Review concluded that antibiotic treatment of BV does not reduce the risk of PTB, regardless of when (before 20 weeks or after 20 weeks of gestation) the treatment is given (48). Some of these organisms possess sialidase activity, which has been associated with an increased risk of PTB (49). Sialidases are hydrolytic enzymes that play a role in down-regulating the innate response by degrading immunoglobin-A (IgA), and it has been used in some diagnostic kits for this reason. Higher LPS concentrations, mostly from *P. bivia* (50), and the concentrations of pro-inflammatory cytokines IL-1β, IL-6, and IL-8 have been found to be elevated in the cervico-vaginal fluid of pregnant women with BV (51). The elevation in vaginal pH above 4.5 is a feature of BV, and this displaces *L. crispatus*, but not *L. iners*, which has adapted to upregulate genes for carbohydrate metabolism (52).

In African American and Hispanic women, a higher abundance of *Mycoplasma* and lower abundance of BVAB3 is associated with an increased risk of PTB in the second trimester (53). This is unlikely due to race *per se*, but rather cultural and social aspects. Other pathogens, such as *Leptotrichia, Sneathia*, BVAB1, and *Mobiluncus* spp. appear in higher abundance prior to 16 weeks gestation in women with a previous history of PTB and who deliver preterm (54). Yet, such findings are not universal, and other studies, albeit small, have reported no difference in the vaginal microbial composition between women who have a spontaneous PTB and those who deliver at term (37, 55).

Future microbiome studies should focus on the functionality of organisms in the vagina, uterus, and perhaps even the placenta (56). This should include the use of metabolomic analysis to help understand how the vaginal microbiome may influence the risk of PTB.

## Role of Immune-Mediators in PTL

The balance of pro and anti-inflammatory cytokines, produced by CD4+ T helper (Th) cells, is important in predicting pregnancy outcomes. In early pregnancy, a modest Th1 pro-inflammatory environment promotes successful implantation and placentation (57). As pregnancy progresses, there is a predominance of Th2 anti-inflammatory cytokines including IL-4 and IL-10, which maintain uterine quiescence (57). Disruption of the Th1/Th2 balance favoring the predominance of Th1 pro-inflammatory cytokines such as IL-1, IL-6, and TNFα may be responsible for some cases of PTL (7). Chemokines, such as IL-8, chemokine ligand (CCL)-2, 3, 4, and 5 attract decidual leukocytes and lead to the recruitment of additional pro-inflammatory cytokines that amplify the inflammatory cascade (58, 59). In the choriodecidua, levels of CCL2, 3, 4, and 5 are increased in women undergoing PTL both with and without infection when compared to women at term not in labor (59). In the amniotic fluid, levels of IL-1β, IL-6, IL-8, TNFα, CCL3, 4, and 5 are elevated in women with threatened PTL, especially in the presence of intra-amniotic infection, as are IL-1β, IL-6, IL-8, TNFα, and CCL2 in the cervical fluid (60–65). IL-6 is increased in the umbilical blood of infants born to mothers with chorioamnionitis (65–67). Furthermore, IL-1β, IL-6, and IL-8 concentrations are increased in maternal plasma women with preterm premature rupture of the membranes and chorioamnionitis (64, 68).

Anti-inflammatory cytokines maintain pregnancy quiescence by inhibiting the production of pro-inflammatory cytokines and PGs (69, 70). IL-10 expression in the placenta is lower in women who give birth preterm with chorioamnionitis compared to samples obtained from women who underwent elective terminations in their second trimester of pregnancy (71). The same has been observed in women in term labor with chorioamnionitis compared to women at term not in labor (71). Amniotic fluid concentrations of IL-10 are not different between preterm and term delivery, while cervico-vaginal levels of IL-4 and IL-10 are often below the level of detection using current assays (72, 73). Data regarding the role of maternal plasma IL-10 in mediating PTB remain conflicting. Some studies report decreased plasma IL-10 concentrations with PTB compared to term (1), whereas others have found an association between elevated plasma IL-10 with an increased risk of pre-eclampsia or intrauterine growth restriction, which may in turn lead to PTB (74). Overall, the positive and negative predictive values of any single specific cytokine or chemokine for PTB is limited (75) although the examination of interactions with a multifactor dimensionality reduction analysis between multiple cytokines within the maternal–fetal compartments, rather than a single cytokine, may better predict the risk of PTB (76).

## Prebiotics and Probiotics for Prevention of PTB

Prebiotics are indigestible food ingredients such as dietary fiber, resistant starch, and oligosaccharides. They confer health benefits by “causing significant changes in the composition of the gut microflora with increased and reduced numbers of potentially health-promoting bacteria and potentially harmful species, respectively” (77, 78). The prebiotics galacto-oligosaccharide (GOS), fructo-oligosaccharides (FOS), and lactulose have been shown to provide substrates for the growth of lactobacilli and bifidobacteria, suggesting that they may contribute to the beneficial effects of probiotics. Prebiotics also possess immune-regulatory functions (79–81) and in particular immune-saccharides are known to induce activation of the innate immune system (81). Prebiotic FOS increases the level of IL-27 concentrations in human milk, which may help prevent the onset of allergic disorders in their children (82). There is anecdotal evidence to suggest that prebiotic-containing food may reduce the risk of PTB (83). Of interest, one study reported that dried fruits and garlic that contained antimicrobial and prebiotic compounds were associated with a reduced risk of spontaneous PTB (84).

Probiotics are defined as “live microorganisms, which when administered in adequate amounts, confer a health benefit on the host” (85). A number of meta-analyses of clinical trials with probiotics have confirmed that probiotics are both safe and effective for the treatment and/or prevention of numerous infectious and/or inflammatory diseases (86–89). *Lactobacillus* and *Bifidobacterium* are the most commonly studied probiotics. Supplementation with *Bifidobacterium lactis* in preterm infants reduces pathogenic *Enterobacteriaceae* and *Clostridium spp*. counts (90). Bifidobacteria are present in large abundance in the intestinal flora, but they can also be detected in the vagina. Probiotic lactobacilli play a potential beneficial role in human reproduction and maintenance of healthy urinary and reproductive tracts (91).

The use of antibiotics to treat BV in non-pregnant and pregnant women remains the method of choice, unchanged in many decades, and still too often ineffective. Metronidazole and clindamycin, by far the most used agents, do not restore vaginal lactobacilli abundance, which may account for relapses in some women; and prolonged use promotes the development of drug resistance (27, 92). The need for new treatment for BV that restores microbiota homeostasis and acidity without undesirable side effects has led investigators and patients to study probiotics. Human studies have provided evidence that probiotic lactobacilli can reduce BV recurrence and increase lactobacilli abundance in the vagina of pregnant and non-pregnant women (93–95). The use of lactobacilli as an adjuvant therapy has also shown promise in lowering BV recurrence rates (92). Indeed, the adjunctive use of *L. rhamnosus* GR-1 and *L. reuteri* RC-14 with metronidazole has been shown to improve actual cure of BV (96, 97).

Probiotic intervention in pregnancy is generally acceptable with good compliance among pregnant women (98). A recent meta-analysis of randomized clinical trials demonstrated that the use of probiotics *Lactobacillus* and *Bifidobacterium* during pregnancy had no effect on the incidence of Cesarean section, birth weight, or gestational age (99).

Oral administration of 10^9^–10^11^ colony-forming units (cfu) of lactobacilli is the standard dose believed to be required for passage through the intestine and subsequent improvement of gut and vaginal health (27, 93, 100, 101). There are many variables that influence vaginal colonization by lactobacilli including glycogen level, substances used in vaginal washing, the use of antibiotics, and the ability of lactobacilli to produce substances such as hydrogen peroxide (102–104). Bodean et al. (92) reported that oral administration of *L. acidophilus* and *L. bifidus* was more effective than the vaginal route in reducing BV occurrence in antibiotic-treated non-pregnant women. However, the probiotic composition of the oral capsule was different from the vaginal capsule (*L. rhamnosus, L. acidophilus, S. thermophilus*, and *L. bulgaricus*) in that study, and the mechanism seems unclear. Furthermore, the treatment duration was longer for patients who received the oral capsule than those who received vaginal capsules (92). An advantage of the oral route is that it may reduce pathogen ascendance from the rectum to perineum and vagina, while a concern of the intravaginal approach for some women may be the more invasive instillation of microbes.

A number of mechanisms whereby lactobacilli defend against pathogens in the vaginal environment have been described, albeit mostly from *in vitro* studies. These include the production of antimicrobial substances, competitive exclusion with pathogenic bacteria and fungi, acidification of the vaginal area, and modulation of the immune system (40). Endogenous lactobacilli maintain the vaginal pH <4.5 by metabolizing glycogen secreted by vaginal mucosal epithelia and produce lactic acid, which is a potent microbicide against potential reproductive tract infections (105, 106). The acidic environment of a healthy vagina creates a hostile environment for BV-associated pathogens while favoring lactobacilli growth (105, 107). It may also help to prevent viruses, such as HIV, from infecting the host (108, 109).

The anti-inflammatory property of lactobacilli has been shown to be important in the control of mucosal and systemic inflammation (110). *L. rhamnosus* GR-1 supernatant (GR-1 SN) enhances IL-10 and colony-stimulating factor 3 (CSF3) production in mouse macrophages (111). In primary human placental trophoblast cells, GR-1 SN increases IL-10 and CSF3 production via JAK/STAT and MAPK pathways, down-regulates LPS-induced TNFα output through c-Jun-N-terminal kinases (JNKs) inhibition, and increases the expression of the PG metabolizing enzyme PGDH in a sex-dependent fashion (112–114). When administered intra-peritoneally to pregnant mice, GR-1 SN reduces LPS-induced PTB in association with a decrease in pro-inflammatory cytokines and an increase in anti-inflammatory cytokines in maternal plasma and the amniotic fluid (115).

The effect of lactobacilli on the immune system and their vaginal colonization ability can be species/strain specific. In the mouse gut, *L. plantarum* and *L. rhamnosus* GG exacerbate inflammation and the development of dextran sulfate sodium (DSS)-induced colitis while *L. paracasei* is protective (116). In the human vagina, *L. rhamnosus* GR-1 and *L. reuteri* RC-14 but not the intestinal probiotic *L. rhamnosus* GG persist up to 19 days (117). Intra-vaginal instillation of *L. rhamnosus* GR-1 has been shown to upregulate some antimicrobial activity in premenopausal women (118). A combination of *B. bifidum, B. infantis, L. acidophilus, L. casei, L. salivarius*, and *Lactococcus lactis* has been reported to provide a wider antimicrobial spectrum, better stimulation of IL-10 production, and suppression of pro-inflammatory cytokines in cultured human peripheral blood mononuclear cells compared to the individual strains (119). A combination of the bacteriocin-like inhibitory substances (BLIS) from the *L. rhamnosus L60* and *L. fermentum L23* can reduce the growth of group B streptococcal isolates obtained from pregnant women more effectively than each *Lactobacillus* strain alone (120).

Lipoteichoic acid (LTA) on the cell surface of lactobacilli can also stimulate macrophages to secrete immune-mediators. Improved anti-inflammatory activity in a murine model of colitis *in vivo* has been observed when LTA is removed or substituted (121–123). *L. rhamnosus* GR-1 supernatant reduces LPS-induced PTB and associated systemic and intrauterine inflammatory cytokines in pregnant mice (115). The supernatant of lactobacilli also has anti-inflammatory properties in cultured human placental trophoblast cells, decidual cells, monocytes, and macrophages (112–114, 124, 125). In human decidual cells challenged with *E. coli*, supernatant of *L. rhamnosus* CNCM I-4036 was found to be more effective than the live bacteria counterpart in the suppression of pro-inflammatory cytokine production (126). These studies imply that administration of supernatant from lactobacilli may promote desirable effects and represent an alternative for the prevention and/or treatment of inflammatory disorders such as some cases of PTB. The identification of these bioactive metabolite(s) remains to be achieved.

Future clinical studies should consider not only the sample size and design but also the appropriate probiotic strain(s), dose and duration of treatment, and route of administration. Until a sufficiently large study is performed in which the rate of PTB is high enough to note a reduction due to an intervention (127), we can only say that currently, the administration of a few probiotic strains is safe for use in pregnancy and shows promise in conferring health benefits, of which potentially reducing the risk of PTB is one.

## Conflict of Interest Statement

The Guest Associate Editor, Jeffrey Keelan, declares that despite being affiliated to the same institution as author, John R. G. Challis, there has been no conflict of interest during the review and handling of this manuscript. The authors declare that the research was conducted in the absence of any commercial or financial relationships that could be construed as a potential conflict of interest.

## References

[1] Makhseed M Raghupathy R El-Shazly S Azizieh F Al-Harmi JA Al-Azemi MM. Pro-inflammatory maternal cytokine profile in preterm delivery. Am J Reprod Immunol (2003) 49:308–18. 10.1034/j.1600-0897.2003.00038.x 12854735

[2] Goldenberg RL Culhane JF Iams JD Romero R Epidemiology and causes of preterm birth. Lancet (2008) 371:75–84 10.1016/S0140-6736(08)60074-4 18177778 PMC7134569

[3] Goldenberg RL Hauth JC Andrews WW Intrauterine infection and preterm delivery. N Engl J Med (2000) 342:1500–7 10.1056/NEJM200005183422007 10816189

[4] Timmons BC Reese J Socrate S Ehinger N Paria BC Milne GL Prostaglandins are essential for cervical ripening in LPS-mediated preterm birth but not term or antiprogestin-driven preterm ripening. Endocrinology (2014) 155:287–98. 10.1210/en.2013-1304 24189143 PMC3868800

[5] Shoji T Yoshida S Mitsunari M Miyake N Tsukihara S Iwabe T Involvement of p38 MAP kinase in lipopolysaccharide-induced production of pro- and anti-inflammatory cytokines and prostaglandin E(2) in human choriodecidua. J Reprod Immunol (2007) 75:82–90. 10.1016/j.jri.2007.05.002 17617469

[6] Romero R Durum S Dinarello CA Oyarzun E Hobbins JC Mitchell MD Interleukin-1 stimulates prostaglandin biosynthesis by human amnion. Prostaglandins (1989) 37:13–22 10.1016/0090-6980(89)90028-2 2785698

[7] Challis JR Lockwood CJ Myatt L Norman JE Strauss JF III Petraglia F Inflammation and pregnancy. Reprod Sci (2009) 16:206–15 10.1177/1933719108329095 19208789

[8] Olgun NS Reznik SE. The matrix metalloproteases and endothelin-1 in infection-associated preterm birth. Obstet Gynecol Int (2010) 2010:657039. 10.1155/2010/657039 20706662 PMC2913859

[9] Maymon E Romero R Pacora P Gomez R Mazor M Edwin S A role for the 72 kDa gelatinase (MMP-2) and its inhibitor (TIMP-2) in human parturition, premature rupture of membranes and intraamniotic infection. J Perinat Med (2001) 29:308–16. 10.1515/JPM.2001.044 11565199

[10] Fujimoto T Parry S Urbanek M Sammel M Macones G Kuivaniemi H A single nucleotide polymorphism in the matrix metalloproteinase-1 (MMP-1) promoter influences amnion cell MMP-1 expression and risk for preterm premature rupture of the fetal membranes. J Biol Chem (2002) 277:6296–302. 10.1074/jbc.M107865200 11741975

[11] Park KH Chaiworapongsa T Kim YM Espinoza J Yoshimatsu J Edwin S Matrix metalloproteinase 3 in parturition, premature rupture of the membranes, and microbial invasion of the amniotic cavity. J Perinat Med (2003) 31:12–22. 10.1515/JPM.2003.002 12661139

[12] Tency I. Inflammatory response in maternal serum during preterm labour. Facts Views Vis Obgyn (2014) 6:19–30. 25009722 PMC4085999

[13] Yoon BH Oh SY Romero R Shim SS Han SY Park JS An elevated amniotic fluid matrix metalloproteinase-8 level at the time of mid-trimester genetic amniocentesis is a risk factor for spontaneous preterm delivery. Am J Obstet Gynecol (2001) 185:1162–7. 10.1067/mob.2001.117678 11717651

[14] Gotsch F Romero R Kusanovic JP Mazaki-Tovi S Pineles BL Erez O The fetal inflammatory response syndrome. Clin Obstet Gynecol (2007) 50:652–83 10.1097/GRF.0b013e31811ebef6 17762416

[15] Buhimschi CS Dulay AT Abdel-Razeq S Zhao G Lee S Hodgson EJ Fetal inflammatory response in women with proteomic biomarkers characteristic of intra-amniotic inflammation and preterm birth. BJOG (2009) 116:257–67. 10.1111/j.1471-0528.2008.01925.x 18947340 PMC3791329

[16] Pacora P Chaiworapongsa T Maymon E Kim YM Gomez R Yoon BH Funisitis and chorionic vasculitis: the histological counterpart of the fetal inflammatory response syndrome. J Matern Fetal Neonatal Med (2002) 11:18–25. 10.1080/jmf.11.1.18.25 12380603

[17] Pavcnik-Arnol M Lucovnik M Kornhauser-Cerar L Premru-Srsen T Hojker S Derganc M Lipopolysaccharide-binding protein as marker of fetal inflammatory response syndrome after preterm premature rupture of membranes. Neonatology (2014) 105:121–7. 10.1159/000356735 24335151

[18] Goldenberg RL Andrews WW Goepfert AR Faye-Petersen O Cliver SP Carlo WA The Alabama Preterm Birth Study: umbilical cord blood *Ureaplasma urealyticum* and *Mycoplasma hominis* cultures in very preterm newborn infants. Am J Obstet Gynecol (2008) 198: 43.e1–5. 10.1016/j.ajog.2007.07.033 18166302 PMC2278008

[19] Gravett MG Hitti J Hess DL Eschenbach DA. Intrauterine infection and preterm delivery: evidence for activation of the fetal hypothalamic-pituitary-adrenal axis. Am J Obstet Gynecol (2000) 182:1404–13. 10.1067/mob.2000.106180 10871456

[20] Challis JR Sloboda DM Alfaidy N Lye SJ Gibb W Patel FA Prostaglandins and mechanisms of preterm birth. Reproduction (2002) 124(1):1–17 10.1530/rep.0.1240001 12090913

[21] Farage M Maibach H. Lifetime changes in the vulva and vagina. Arch Gynecol Obstet (2006) 273:195–202.1. 10.1007/s00404-005-0079-x 16208476

[22] Spear GT French AL Gilbert D Zariffard MR Mirmonsef P Sullivan TH Human alpha-amylase present in lower-genital-tract mucosal fluid processes glycogen to support vaginal colonization by *Lactobacillus*. J Infect Dis (2014) 210(7):1019–28. 10.1093/infdis/jiu231 24737800 PMC4168305

[23] Romero R Hassan SS Gajer P Tarca AL Fadrosh DW Nikita L The composition and stability of the vaginal microbiota of normal pregnant women is different from that of non-pregnant women. Microbiome (2014) 2:4 10.1186/2049-2618-2-4 24484853 PMC3916806

[24] Cauci S Driussi S De Santo D Penacchioni P Iannicelli T Lanzafame P Prevalence of bacterial vaginosis and vaginal flora changes in peri- and postmenopausal women. J Clin Microbiol (2002) 40:2147–52. 10.1128/JCM.40.6.2147-2152.2002 12037079 PMC130764

[25] Gupta S Kumar N Singhal N Kaur R Manektala U. Vaginal microflora in postmenopausal women on hormone replacement therapy. Indian J Pathol Microbiol (2006) 49:457–61. 17001923

[26] Hummelen R Macklaim JM Bisanz JE Hammond JA McMillan A Vongsa R Vaginal microbiome and epithelial gene array in post-menopausal women with moderate to severe dryness. PLoS One (2011) 6:e26602. 10.1371/journal.pone.0026602 22073175 PMC3206802

[27] Othman M Neilson JP Alfirevic Z. Probiotics for preventing preterm labour. Cochrane Database Syst Rev (2007) 1:CD005941. 10.1002/14651858.CD005941.pub2 17253567 PMC9006117

[28] Aagaard K Riehle K Ma J Segata N Mistretta TA Coarfa C A metagenomic approach to characterization of the vaginal microbiome signature in pregnancy. PLoS One (2012) 7:e36466. 10.1371/journal.pone.0036466 22719832 PMC3374618

[29] Chaban B Links MG Jayaprakash TP Wagner EC Bourque DK Lohn Z Characterization of the vaginal microbiota of healthy Canadian women through the menstrual cycle. Microbiome (2014) 2:23. 10.1186/2049-2618-2-23 25053998 PMC4106219

[30] Lamont RF Sobel JD Akins RA Hassan SS Chaiworapongsa T Kusanovic JP The vaginal microbiome: new information about genital tract flora using molecular based techniques. BJOG (2011) 118:533–49. 10.1111/j.1471-0528.2010.02840.x 21251190 PMC3055920

[31] Verstraelen H Verhelst R Claeys G Temmerman M Vaneechoutte M. Culture-independent analysis of vaginal microflora: the unrecognized association of *Atopobium vaginae* with bacterial vaginosis. Am J Obstet Gynecol (2004) 191:1130–2. 10.1016/j.ajog.2004.04.013 15507931

[32] Fredricks DN Fiedler TL Marrazzo JM. Molecular identification of bacteria associated with bacterial vaginosis. N Engl J Med (2005) 353:1899–911. 10.1056/NEJMoa043802 16267321

[33] Gloor GB Hummelen R Macklaim JM Dickson RJ Fernandes AD MacPhee R Microbiome profiling by illumina sequencing of combinatorial sequence-tagged PCR products. PLoS One (2010) 5:e15406. 10.1371/journal.pone.0015406 21048977 PMC2964327

[34] Walther-Antonio MR Jeraldo P Berg Miller ME Yeoman CJ Nelson KE Wilson BA Pregnancy’s stronghold on the vaginal microbiome. PLoS One (2014) 9:e98514. 10.1371/journal.pone.0098514 24896831 PMC4045671

[35] Ravel J Gajer P Abdo Z Schneider GM Koenig SS McCulle SL Vaginal microbiome of reproductive-age women. Proc Natl Acad Sci U S A (2011) 108(Suppl 1):4680–7 10.1073/pnas.1002611107 20534435 PMC3063603

[36] Verstraelen H Verhelst R Claeys G De Backer E Temmerman M Vaneechouttee M Longitudinal analysis of the vaginal microflora in pregnancy suggests that *L. crispatus* promotes the stability of the normal vaginal microflora and that *L. gasseri* and/or *L. iners* are more conducive to the occurrence of abnormal vaginal microflora. BMC Microbiol (2009) 9:116. 10.1186/1471-2180-9-116 19490622 PMC2698831

[37] Romero R Hassan SS Gajer P Tarca AL Fadrosh DW Bieda J The vaginal microbiota of pregnant women who subsequently have spontaneous preterm labor and delivery and those with a normal delivery at term. Microbiome (2014) 2:18. 10.1186/2049-2618-2-18 24987521 PMC4066267

[38] Schwebke JR Muzny CA Josey WE. Role of *Gardnerella vaginalis* in the pathogenesis of bacterial vaginosis: a conceptual model. J Infect Dis (2014) 210:338–43. 10.1093/infdis/jiu089 24511102

[39] van de Wijgert JH Borgdorff H Verhelst R Crucitti T Francis S Verstraelen H The vaginal microbiota: what have we learned after a decade of molecular characterization? PLoS One (2014) 9:e105998. 10.1371/journal.pone.0105998 25148517 PMC4141851

[40] Donders GG Van Calsteren K Bellen G Reybrouck R Van den Bosch T Riphagen I Predictive value for preterm birth of abnormal vaginal flora, bacterial vaginosis and aerobic vaginitis during the first trimester of pregnancy. BJOG (2009) 116:1315–24. 10.1111/j.1471-0528.2009.02237.x 19538417

[41] Reid G Bocking A. The potential for probiotics to prevent bacterial vaginosis and preterm labor. Am J Obstet Gynecol (2003) 189:1202–8. 10.1067/S0002-9378(03)00495-2 14586379

[42] Nugent RP Krohn MA Hillier SL. Reliability of diagnosing bacterial vaginosis is improved by a standardized method of gram stain interpretation. J Clin Microbiol (1991) 29:297–301. 1706728 10.1128/jcm.29.2.297-301.1991 PMC269757

[43] Hummelen R Fernandes AD Macklaim JM Dickson RJ Changalucha J Gloor GB Deep sequencing of the vaginal microbiota of women with HIV. PLoS One (2010) 5:e12078. 10.1371/journal.pone.0012078 20711427 PMC2920804

[44] Jakobsson T Forsum U *Lactobacillus iners*: a marker of changes in the vaginal flora? J Clin Microbiol (2007) 45:3145 10.1128/JCM.00558-07 17652481 PMC2045263

[45] Menard JP Fenollar F Henry M Bretelle F Raoult D. Molecular quantification of *Gardnerella vaginalis* and *Atopobium vaginae* loads to predict bacterial vaginosis. Clin Infect Dis (2008) 47:33–43. 10.1086/588661 18513147

[46] Ugwumadu AH Bacterial vaginosis in pregnancy. Curr Opin Obstet Gynecol (2002) 14:115–8 10.1086/588661 11914687

[47] McDonald HM Brocklehurst P Gordon A Antibiotics for treating bacterial vaginosis in pregnancy. Cochrane Database Syst Rev (2007) 1:CD000262 10.1002/14651858.CD000262.pub3 17253447 PMC4164464

[48] Brocklehurst P Gordon A Heatley E Milan SJ Antibiotics for treating bacterial vaginosis in pregnancy. Cochrane Database Syst Rev (2013) 1:CD000262 10.1002/14651858.CD000262.pub4 23440777 PMC11307253

[49] Smayevsky J Canigia LF Lanza A Bianchini H. Vaginal microflora associated with bacterial vaginosis in nonpregnant women: reliability of sialidase detection. Infect Dis Obstet Gynecol (2001) 9:17–22. 10.1155/S1064744901000047 11368254 PMC1784631

[50] Aroutcheva A Ling Z Faro S. *Prevotella bivia* as a source of lipopolysaccharide in the vagina. Anaerobe (2008) 14:256–60. 10.1016/j.anaerobe.2008.08.002 18849004 PMC2651005

[51] Mitchell C Marrazzo J. Bacterial vaginosis and the cervicovaginal immune response. Am J Reprod Immunol (2014) 71:555–63. 10.1111/aji.12264 24832618 PMC4128638

[52] Macklaim JM Fernandes AD Di Bella JM Hammond JA Reid G Gloor GB. Comparative meta RNA-seq of the vaginal microbiota and differential expression by *Lactobacillus iners* in health and dysbiosis. Microbiome (2013) 1:12. 10.1186/2049-2618-1-12 24450540 PMC3971606

[53] Wen A Srinivasan U Goldberg D Owen J Marrs CF Misra D Selected vaginal bacteria and risk of preterm birth: an ecological perspective. J Infect Dis (2014) 209:1087–94. 10.1093/infdis/jit632 24273044 PMC3952673

[54] Nelson DB Hanlon A Nachamkin I Haggerty C Mastrogiannis DS Liu C Early pregnancy changes in bacterial vaginosis-associated bacteria and preterm delivery. Paediatr Perinat Epidemiol (2014) 28:88–96. 10.1111/ppe.12106 24405280 PMC4031320

[55] Hyman RW Fukushima M Jiang H Fung E Rand L Johnson B Diversity of the vaginal microbiome correlates with preterm birth. Reprod Sci (2014) 21:32–40. 10.1177/1933719113488838 23715799 PMC3857766

[56] Aagaard K Ma J Antony KM Ganu R Petrosino J Versalovic J The placenta harbors a unique microbiome. Sci Transl Med (2014) 6:237ra265. 10.1126/scitranslmed.3008599 24848255 PMC4929217

[57] Wilczynski JR. Th1/Th2 cytokines balance – yin and yang of reproductive immunology. Eur J Obstet Gynecol Reprod Biol (2005) 122:136–43. 10.1016/j.ejogrb.2005.03.008 15893871

[58] Esplin MS Peltier MR Hamblin S Smith S Fausett MB Dildy GA Monocyte chemotactic protein-1 expression is increased in human gestational tissues during term and preterm labor. Placenta (2005) 26:661–71. 10.1016/j.placenta.2004.09.012 16085045

[59] Hamilton SA Tower CL Jones RL. Identification of chemokines associated with the recruitment of decidual leukocytes in human labour: potential novel targets for preterm labour. PLoS One (2013) 8:e56946. 10.1371/journal.pone.0056946 23451115 PMC3579936

[60] El-Bastawissi AY Williams MA Riley DE Hitti J Krieger JN Amniotic fluid interleukin-6 and preterm delivery: a review. Obstet Gynecol (2000) 95:1056–64 10.1016/S0029-7844(00)00875-9 10808034

[61] Hitti J Hillier SL Agnew KJ Krohn MA Reisner DP Eschenbach DA Vaginal indicators of amniotic fluid infection in preterm labor. Obstet Gynecol (2001) 97:211–9. 10.1016/S0029-7844(00)01146-7 11165584

[62] Jun JK Yoon BH Romero R Kim M Moon JB Ki SH Interleukin 6 determinations in cervical fluid have diagnostic and prognostic value in preterm premature rupture of membranes. Am J Obstet Gynecol (2000) 183:868–73. 10.1067/mob.2000.109034 11035328

[63] Coultrip LL Lien JM Gomez R Kapernick P Khoury A Grossman JH The value of amniotic fluid interleukin-6 determination in patients with preterm labor and intact membranes in the detection of microbial invasion of the amniotic cavity. Am J Obstet Gynecol (1994) 171:901–11 10.1016/S0002-9378(94)70057-5 7943100

[64] von Minckwitz G Grischke EM Schwab S Hettinger S Loibl S Aulmann M Predictive value of serum interleukin-6 and -8 levels in preterm labor or rupture of the membranes. Acta Obstet Gynecol Scand (2000) 79:667–72. 10.1034/j.1600-0412.2000.079008667.x 10949232

[65] Holst RM Hagberg H Wennerholm UB Skogstrand K Thorsen P Jacobsson B. Prediction of microbial invasion of the amniotic cavity in women with preterm labour: analysis of multiple proteins in amniotic and cervical fluids. BJOG (2011) 118:240–9. 10.1111/j.1471-0528.2010.02765.x 21054762

[66] Chaiworapongsa T Romero R Kim JC Kim YM Blackwell SC Yoon BH Evidence for fetal involvement in the pathologic process of clinical chorioamnionitis. Am J Obstet Gynecol (2002) 186:1178–82. 10.1067/mob.2002.124042 12066094

[67] Holst RM Mattsby-Baltzer I Wennerholm UB Hagberg H Jacobsson B. Interleukin-6 and interleukin-8 in cervical fluid in a population of Swedish women in preterm labor: relationship to microbial invasion of the amniotic fluid, intra-amniotic inflammation, and preterm delivery. Acta Obstet Gynecol Scand (2005) 84:551–7. 10.1111/j.0001-6349.2005.00708.x 15901266

[68] Torbe A Czajka R Kordek A Rzepka R Kwiatkowski S Rudnicki J. Maternal serum proinflammatory cytokines in preterm labor with intact membranes: neonatal outcome and histological associations. Eur Cytokine Netw (2007) 18:102–7. 10.1684/ecn.2007.0092 17594943

[69] Bowen JM Chamley L Mitchell MD Keelan JA. Cytokines of the placenta and extra placental membranes: biosynthesis, secretion and roles in establishment of pregnancy in women. Placenta (2002) 23:239–56. 10.1053/plac.2001.0781 11969335

[70] Keelan JA Blumenstein M Helliwell RJ Sato TA Marvin KW Mitchell MD Cytokines, prostaglandins and parturition – a review. Placenta (2003) 24(Suppl A):S33–46 10.1053/plac.2002.0948 12842412

[71] Hanna N Bonifacio L Weinberger B Reddy P Murphy S Romero R Evidence for interleukin 10-mediated inhibition of cyclo-oxygenase-2 expression and prostaglandin production in preterm human placenta. Am J Reprod Immunol (2006) 55:19–27. 10.1111/j.1600-0897.2005.00342.x 16364008

[72] Puchner K Iavazzo C Gourgiotis D Boutsikou M Baka S Hassiakos D Mid-trimester amniotic fluid interleukins (IL-1beta, IL-10 and IL-18) as possible predictors of preterm delivery. In vivo (2011) 25:141–8. 21282748

[73] Vogel I Goepfert AR Thorsen P Skogstrand K Hougaard DM Curry AH Early second-trimester inflammatory markers and short cervical length and the risk of recurrent preterm birth. J Reprod Immunol (2007) 75:133–40. 10.1016/j.jri.2007.02.008 17442403

[74] Ferguson KK McElrath TF Chen YH Mukherjee B Meeker JD. Longitudinal profiling of inflammatory cytokines and C-reactive protein during uncomplicated and preterm pregnancy. Am J Reprod Immunol (2014) 72:326–36. 10.1111/aji.12265 24807462 PMC4573571

[75] Menon R Torloni MR Voltolini C Torricelli M Merialdi M Betran AP Biomarkers of spontaneous preterm birth: an overview of the literature in the last four decades. Reprod Sci (2011) 18:1046–70. 10.1177/1933719111415548 22031189

[76] Bhat G Williams SM Saade GR Menon R. Biomarker interactions are better predictors of spontaneous preterm birth. Reprod Sci (2014) 21:340–50. 10.1177/1933719113497285 23885102

[77] Gibson GR Probert HM Loo JV Rastall RA Roberfroid MB. Dietary modulation of the human colonic microbiota: updating the concept of prebiotics. Nutr Res Rev (2004) 17:259–75. 10.1079/NRR200479 19079930

[78] Roberfroid M Prebiotics: the concept revisited. J Nutr (2007) 137:830s–7s. 17311983 10.1093/jn/137.3.830S

[79] Watson D O’Connell Motherway M Schoterman MH van Neerven RJ Nauta A van Watson D Selective carbohydrate utilization by lactobacilli and bifidobacteria. J Appl Microbiol (2013) 114:1132–46. 10.1111/jam.12105 23240984

[80] Ben XM Li J Feng ZT Shi SY Lu YD Chen R Low level of galacto-oligosaccharide in infant formula stimulates growth of intestinal bifidobacteria and Lactobacilli. World J Gastroenterol (2008) 14:6564–8. 10.3748/wjg.14.6564 19030213 PMC2773347

[81] Song SK Beck BR Kim D Park J Kim J Kim HD Prebiotics as immunostimulants in aquaculture: a review. Fish Shellfish Immunol (2014) 40:40–8. 10.1016/j.fsi.2014.06.016 24973515

[82] Kubota T Shimojo N Nonaka K Yamashita M Ohara O Igoshi Y Prebiotic consumption in pregnant and lactating women increases IL-27 expression in human milk. Br J Nutr (2014) 111:625–32. 10.1017/S0007114513003036 24073873

[83] Otsuki K Tokunaka M Oba T Nakamura M Shirato N Okai T. Administration of oral and vaginal prebiotic lactoferrin for a woman with a refractory vaginitis recurring preterm delivery: appearance of *lactobacillus* in vaginal flora followed by term delivery. J Obstet Gynaecol Res (2014) 40:583–5. 10.1111/jog.12171 24118573

[84] Myhre R Brantsaeter AL Myking S Eggesbo M Meltzer HM Haugen M Intakes of garlic and dried fruits are associated with lower risk of spontaneous preterm delivery. J Nutr (2013) 143:1100–8. 10.3945/jn.112.173229 23700347 PMC3681545

[85] FAO/WHO. Joint FAO/WHO Expert Consultation on Evaluation of Health and Nutritional Properties of Probiotics in Food. London, ON (2001). Available from: ftp://ftp.fao.org/docrep/fao/009/a0512e/a0512e00.pdf.

[86] Chen AC Chung MY Chang JH Lin HC. Pathogenesis implication for necrotizing enterocolitis prevention in preterm very-low-birth-weight infants. J Pediatr Gastroenterol Nutr (2014) 58:7–11. 10.1097/MPG.0b013e3182a7dc74 24378520

[87] Goldenberg JZ Ma SS Saxton JD Martzen MR Vandvik PO Thorlund K Probiotics for the prevention of *Clostridium difficile*-associated diarrhea in adults and children. Cochrane Database Syst Rev (2013) 5:CD006095. 10.1002/14651858.CD006095.pub3 23728658

[88] Yang Y Guo Y Kan Q Zhou XG Zhou XY Li Y A meta-analysis of probiotics for preventing necrotizing enterocolitis in preterm neonates. Braz J Med Biol Res (2014) 47:804–10 10.1590/1414-431X20143857 25098619 PMC4143209

[89] Grin PM Kowalewska PM Alhazzan W Fox-Robichaud AE. *Lactobacillus* for preventing recurrent urinary tract infections in women: meta-analysis. Can J Urol (2013) 20:6607–14. 23433130

[90] Szajewska H Guandalini S Morelli L Van Goudoever JB Walker A. Effect of *Bifidobacterium animalis subsp lactis* supplementation in preterm infants: a systematic review of randomized controlled trials. J Pediatr Gastroenterol Nutr (2010) 51:203–9. 10.1097/MPG.0b013e3181dc0d93 20543719 PMC4507410

[91] Reid G Brigidi P Burton JP Contractor N Duncan S Fargier E Microbes central to human reproduction. Am J Reprod Immunol (2014) 73(1):1–11 10.1111/aji.12319 25250861 PMC4282787

[92] Bodean O Munteanu O Cirstoiu C Secara D Cirstoiu M. Probiotics-a helpful additional therapy for bacterial vaginosis. J Med Life (2013) 6:434–6. 24868256 PMC4034315

[93] Homayouni A Bastani P Ziyadi S Mohammad-Alizadeh-Charandabi S Ghalibaf M Mortazavian AM Effects of probiotics on the recurrence of bacterial vaginosis: a review. J Low Genit Tract Dis (2014) 18:79–86. 10.1097/LGT.0b013e31829156ec 24299970

[94] Nishijima K Shukunami K Kotsuji F Probiotics affects vaginal flora in pregnant women, suggesting the possibility of preventing preterm labor. J Clin Gastroenterol (2005) 39:447–8 10.1097/01.mcg.0000159269.58480.4b 15815217

[95] Reid G Charbonneau D Erb J Kochanowski B Beuerman D Poehner R Oral use of *Lactobacillus rhamnosus* GR-1 and *L. fermentum* RC-14 significantly alters vaginal flora: randomized, placebo-controlled trial in 64 healthy women. FEMS Immunol Med Microbiol (2003) 35:131–4. 10.1016/S0928-8244(02)00465-0 12628548

[96] Martinez RC Franceschini SA Patta MC Quintana SM Gomes BC De Martinis EC Improved cure of bacterial vaginosis with single dose of tinidazole (2 g), *Lactobacillus rhamnosus* GR-1, and *Lactobacillus reuteri* RC-14: a randomized, double-blind, placebo-controlled trial. Can J Microbiol (2009) 55:133–8. 10.1139/w08-102 19295645

[97] Anukam K Osazuwa E Ahonkhai I Ngwu M Osemene G Bruce AW Augmentation of antimicrobial metronidazole therapy of bacterial vaginosis with oral probiotic *Lactobacillus rhamnosus* GR-1 and *Lactobacillus reuteri* RC-14: randomized, double-blind, placebo controlled trial. Microbes Infect (2006) 8:1450–4. 10.1016/j.micinf.2006.01.003 16697231

[98] Lindsay KL Brennan L McAuliffe FM Acceptability of and compliance with a probiotic capsule intervention in pregnancy. Int J Gynaecol Obstet (2014) 125:279–80 10.1016/j.ijgo.2014.01.004 24636629

[99] Dugoua JJ Machado M Zhu X Chen X Koren G Einarson TR Probiotic safety in pregnancy: a systematic review and meta-analysis of randomized controlled trials of *Lactobacillus*, *Bifidobacterium*, and *Saccharomyces* spp. J Obstet Gynaecol Can (2009) 31:542–52. 19646321 10.1016/S1701-2163(16)34218-9

[100] Reid G Beuerman D Heinemann C Bruce AW. Probiotic *Lactobacillus* dose required to restore and maintain a normal vaginal flora. FEMS Immunol Med Microbiol (2001) 32:37–41. 10.1111/j.1574-695X.2001.tb00531.x 11750220

[101] Morelli L Zonenenschain D Del Piano M Cognein P. Utilization of the intestinal tract as a delivery system for urogenital probiotics. J Clin Gastroenterol (2004) 38(6 Suppl):S107–10. 10.1097/01.mcg.0000128938.32835.98 15220672

[102] Vallor AC Antonio MA Hawes SE Hillier SL. Factors associated with acquisition of, or persistent colonization by, vaginal lactobacilli: role of hydrogen peroxide production. J Infect Dis (2001) 184(11):1431–6. 10.1086/324445 11709785

[103] Baeten JM Hassan WM Chohan V Richardson BA Mandaliya K Ndinya-Achola JO Prospective study of correlates of vaginal *Lactobacillus* colonisation among high-risk HIV-1 seronegative women. Sex Transm Infect (2009) 85(5):348–53. 10.1136/sti.2008.035451 19329442 PMC2837477

[104] Mirmonsef P Hotton AL Gilbert D Burgad D Landay A Weber KM Free glycogen in vaginal fluids is associated with *Lactobacillus* colonization and low vaginal pH. PLoS One (2014) 9(7):e102467. 10.1371/journal.pone.0102467 25033265 PMC4102502

[105] Donati L Di Vico A Nucci M Quagliozzi L Spagnuolo T Labianca A Vaginal microbial flora and outcome of pregnancy. Arch Gynecol Obstet (2010) 281:589–600. 10.1007/s00404-009-1318-3 19967381

[106] O’Hanlon DE Moench TR Cone RA. Vaginal pH and microbicidal lactic acid when lactobacilli dominate the microbiota. PLoS One (2013) 8:e80074. 10.1007/s00404-009-1318-3 24223212 PMC3819307

[107] Borges S Silva J Teixeira P. The role of lactobacilli and probiotics in maintaining vaginal health. Arch Gynecol Obstet (2014) 289:479–89. 10.1007/s00404-013-3064-9 24170161

[108] Cadieux P Burton J Gardiner G Braunstein I Bruce AW Kang CY *Lactobacillus* strains and vaginal ecology. JAMA (2002) 287:1940–1 10.1001/jama.287.15.1935 11960535

[109] Petrova MI van den Broek M Balzarini J Vanderleyden J Lebeer S. Vaginal microbiota and its role in HIV transmission and infection. FEMS Microbiol Rev (2013) 37:762–92. 10.1111/1574-6976.12029 23789590

[110] Kemgang TS Kapila S Shanmugam VP Kapila R. Cross-talk between probiotic lactobacilli and host immune system. J Appl Microbiol (2014) 117:303–19. 10.1111/jam.12521 24738909

[111] Kim SO Sheikh HI Ha SD Martins A Reid G. G-CSF-mediated inhibition of JNK is a key mechanism for *Lactobacillus rhamnosus*-induced suppression of TNF production in macrophages. Cell Microbiol (2006) 8:1958–71. 10.1111/j.1462-5822.2006.00763.x 16889627

[112] Yeganegi M Leung CG Martins A Kim SO Reid G Challis JR *Lactobacillus rhamnosus* GR-1 stimulates colony-stimulating factor 3 (granulocyte) (CSF3) output in placental trophoblast cells in a fetal sex-dependent manner. Biol Reprod (2011) 84:18–25. 10.1095/biolreprod.110.085167 20811016 PMC4480822

[113] Yeganegi M Leung CG Martins A Kim SO Reid G Challis JR *Lactobacillus rhamnosus* GR-1 induced IL-10 production in human placental trophoblast cells involves activation of JAK/STAT and MAPK pathways. Reprod Sci (2010) 17:1043–51. 10.1177/1933719110377237 20858906

[114] Yeganegi M Watson CS Martins A Kim SO Reid G Challis JR Effect of *Lactobacillus rhamnosus* GR-1 supernatant and fetal sex on lipopolysaccharide-induced cytokine and prostaglandin-regulating enzymes in human placental trophoblast cells: implications for treatment of bacterial vaginosis and prevention of preterm labor. Am J Obstet Gynecol (2009) 200(532):e531–8. 10.1016/j.ajog.2008.12.032 19285652

[115] Yang S Li W Challis JR Reid G Kim SO Bocking AD. Probiotic *Lactobacillus rhamnosus* GR-1 supernatant prevents lipopolysaccharide-induced preterm birth and reduces inflammation in pregnant CD-1 mice. Am J Obstet Gynecol (2014) 211: 44.e1–44.e12. 10.1016/j.ajog.2014.01.029 24486224

[116] Mileti E Matteoli G Iliev ID Rescigno M. Comparison of the immunomodulatory properties of three probiotic strains of lactobacilli using complex culture systems: prediction for *in vivo* efficacy. PLoS One (2009) 4:e7056. 10.1371/journal.pone.0007056 19756155 PMC2738944

[117] Gardiner GE Heinemann C Bruce AW Beuerman D Reid G. Persistence of *Lactobacillus fermentum* RC-14 and *Lactobacillus rhamnosus* GR-1 but not *L. rhamnosus* GG in the human vagina as demonstrated by randomly amplified polymorphic DNA. Clin Diagn Lab Immunol (2002) 9:92–6. 10.1128/CDLI.9.1.92-96.2002 11777835 PMC119863

[118] Kirjavainen PK Laine RM Carter D Hammond J-A Reid G Expression of anti microbial defense factors in vaginal mucosa following exposure of *Lactobacillus rhamnosus* GR-1. Int J Probiotics (2008) 3:99–106.

[119] Timmerman HM Niers LE Ridwan BU Koning CJ Mulder L Akkemans LM Design of a multispecies probiotic mixture to prevent infectious complications in critically ill patients. Clin Nutr (2007) 26:450–9. 10.1016/j.clnu.2007.04.008 17544549

[120] Ruiz FO Gerbaldo G Garcia MJ Giordano W Pascual L Barberis IL Synergistic effect between two bacteriocin-like inhibitory substances produced by lactobacilli strains with inhibitory activity for *Streptococcus agalactiae*. Curr Microbiol (2012) 64:349–56. 10.1007/s00284-011-0077-0 22231454

[121] Grangette C Nutten S Palumbo E Morath S Hermann C Dewulf J Enhanced antiinflammatory capacity of a *Lactobacillus plantarum* mutant synthesizing modified teichoic acids. Proc Natl Acad Sci U S A (2005) 102:10321–6. 10.1073/pnas.0504084102 15985548 PMC1177390

[122] Claes IJ Lebeer S Shen C Verhoeven TL Dilissen E De Hertogh G Impact of lipoteichoic acid modification on the performance of the probiotic *Lactobacillus rhamnosus* GG in experimental colitis. Clin Exp Immunol (2010) 162:306–14. 10.1111/j.1365-2249.2010.04228.x 20731672 PMC2996598

[123] Mohamadzadeh M Pfeiler EA Brown JB Zadeh M Gramarossa M Managlia E Regulation of induced colonic inflammation by *Lactobacillus acidophilus* deficient in lipoteichoic acid. Proc Natl Acad Sci U S A (2011) 108(Suppl 1):4623–30 10.1073/pnas.1005066107 21282652 PMC3063598

[124] Li W Yang S Kim SO Reid G Challis JR Bocking AD. Lipopolysaccharide-induced profiles of cytokine, chemokine, and growth factors produced by human decidual cells are altered by *Lactobacillus rhamnosus* GR-1 supernatant. Reprod Sci (2014) 21:939–47. 10.1177/1933719113519171 24429676 PMC4107568

[125] Lin YP Thibodeaux CH Pena JA Ferry GD Versalovic J. Probiotic *Lactobacillus reuteri* suppress proinflammatory cytokines via c-Jun. Inflamm Bowel Dis (2008) 14:1068–83. 10.1002/ibd.20448 18425802

[126] Bermudez-Brito M Munoz-Quezada S Gomez-Llorente C Romero F Gil A. *Lactobacillus rhamnosus* and its cell-free culture supernatant differentially modulate inflammatory biomarkers in *Escherichia coli*-challenged human dendritic cells. Br J Nutr (2014) 111:1727–37. 10.1017/S0007114513004303 24480321

[127] Krauss-Silva L Moreira ME Alves MB Braga A Camacho KG Batista MR A randomised controlled trial of probiotics for the prevention of spontaneous preterm delivery associated with bacterial vaginosis: preliminary results. Trials (2011) 12:239. 10.1186/1745-6215-12-239 22059409 PMC3264514

